# Obstacles for optimal tuberculosis case detection in primary health centers (PHC) in Sidoarjo district, East Java, Indonesia

**DOI:** 10.1186/1472-6963-7-135

**Published:** 2007-08-30

**Authors:** Chatarina U Wahyuni, Budiono, Lutfia Dwi Rahariyani, Muji Sulistyowati, Tety Rachmawati, Djuwari, Sri Yuliwati, Marieke J van der Werf

**Affiliations:** 1 Department of Epidemiology, School of Public Health, Airlangga University, Surabaya, Indonesia; 2 Faculty of Medicine, Airlangga University, Surabaya, Indonesia; 3 Institute of Health Polytechnic, Surabaya, Indonesia; 4 Department of Health Promotion and Behavior, School of Public Health, Airlangga University, Surabaya, Indonesia; 5 Center of Research and Development for Health Systems and Policy, Surabaya, Indonesia; 6 District Health Office of Lamongan, East Java, Indonesia; 7 District Health Office of Sidoarjo, East Java, Indonesia; 8 KNCV Tuberculosis Foundation, The Hague, The Netherlands; 9 CINIMA, Academic Medical Center, University of Amsterdam, The Netherlands

## Abstract

**Background:**

Pulmonary tuberculosis (TB) is a major health problem worldwide. Detection of the most infectious cases of tuberculosis – sputum smear-positive pulmonary cases – by passive case finding is an essential component of TB control. The district of Sidoarjo in East Java reported a low case detection rate (CDR) of 14% in 2003. We evaluated the diagnostic process for TB in primary health care centers (PHC) in Sidoarjo district to assess whether problems in identification of TB suspects or in diagnosing TB patients can explain the low CDR.

**Methods:**

We performed interviews with the staff (general nurse, TB worker, laboratory technician, and head of health center) of the 25 PHCs of Sidoarjo district to obtain information about the knowledge of TB, health education practices, and availability of support services for TB diagnosis. The quality of the laboratory diagnosis was examined by providing 10 slides with a known result to the laboratory technicians for re-examination.

**Results:**

Eighty percent of the nurses and 84% of the TB workers knew that cough >3 weeks can be a symptom of TB. Only 40% of the nurses knew the cause of TB, few could mention complications of TB and none could mention the duration of infectiousness after start of treatment. Knowledge of TB workers was much better. Information about how to produce a good sputum sample was provided to TB suspects by 76% of the nurses and 84% of the TB workers. Only few provided all information. Fifty-five percent of the 11 laboratory technicians correctly identified all positive slides as positive and 45% correctly identified 100% of the negative slides as negative. All TB workers, one general nurses and 32% of the laboratory technicians had received specific training in TB control. There has been no shortage of TB forms and laboratory materials in 96% of the PHCs.

**Conclusion:**

The quality of the diagnostic process for TB at PHC in Sidoarjo district should be improved on all levels. Training in TB control of all general nurses and the laboratory technicians that have not received training would be a good first step to enhance diagnosis of TB and to improve the case detection rate.

## Background

Pulmonary tuberculosis (TB) is a major global health problem, which claims 1.7 million deaths per year [1]. The epidemic situation was so worrisome in some countries that in 1991 the World Health Assembly declared TB a global public health emergency. In 1994, the internationally recommended control strategy DOTS was launched [2]. This strategy recommends passive case detection, diagnosis by smear microscopy and prompt treatment of detected cases. Detection of infectious cases of tuberculosis is an essential component of TB control. By early detection of infectious cases the transmission of tuberculosis in the community can be limited. Furthermore, early detection of cases increases the chance that they can be treated successfully.

Indonesia has adopted the DOTS strategy in 1995. The province of East Java started implementation of the DOTS strategy in 2000 in all districts, reaching a primary health center (PHC) coverage of 100% in 2004. In 2003, the province reported identification of 41,179 TB suspects and registration of 11,533 new smear positive cases [3]. This provided a case detection rate (CDR) of 28%, far from the target of 70%. A low CDR can be the result of an inaccurate too high estimate of the incidence of new smear positive TB. The incidence estimate of Indonesia was obtained from information of local TB prevalence surveys conducted between 1979 and 1982 who were not performed with the aim of obtaining an unbiased national estimate [4,5]. Furthermore, only persons who reported cough ≥ 2 weeks were asked for one sputum sample. The fact that the incidence estimate is obtained from relatively old data that were not collected to obtain an unbiased national estimate and for which an insensitive screening method was used may have resulted in an inaccurate estimate of TB incidence for Indonesia. A low case detection rate can also be observed if patients do not seek care; do not seek care at health care facilities reporting to the National Tuberculosis Program (NTP) but e.g. at private clinics; or if the health care facilities are not able to identify TB suspects or diagnose TB. It is estimated that as many as 5–10% of adults attending outpatient health facilities in developing countries have a persistent cough of more than 2–3 weeks duration and would thus qualify for sputum examination according to the guidelines [6]. In well functioning facilities, staff should therefore spend a significant amount of time on identification and examination of persons with persistent cough and referral for smear examination.

The district of Sidoarjo had a population of 1,629,311 in 2003. The district notified a low number of TB suspects (4,529, 21.4% of the expected 21,181 cases) and sputum smear positive TB patients (292, 13.8% of the expected 2,118 cases) even though having sufficient human resources, a solid recording and reporting system, and an adequate budget for the TB control program (Sidoarjo Health Office, 2003).

In Sidoarjo district there are five private hospitals that have all implemented the DOTS strategy in 2004 for diagnosis and treatment of TB for patients visiting the general policlinic. Method of diagnosis and treatment of patients that visit the specialist polyclinic depends on the doctor.

We decided to evaluate the diagnostic process for tuberculosis and the supporting services in primary health care centers (PHC) in Sidoarjo district to assess whether problems in identification of TB suspects and diagnosing TB patients can explain the low CDR.

## Methods

The Sidoarjo health office employs two health workers (wasors) who supervise the TB control program and one communicable disease center (CDC) section officer who organizes the TB program. There are 25 primary health care centers (PHCs) in Sidoarjo: 9 laboratory referral health centers (Puskesmas Rujukan Mikroskopis = PRM), 2 independent laboratory health centers (Puskesmas Pelaksana Mandiri = PPM) and 14 satellite health centers (Puskesmas Satelit = PS).

**Figure 1:**
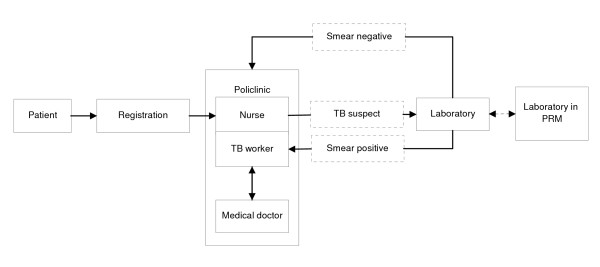
Flowchart of TB services at the primary health centers in Sidoarjo district.

Patients that visit the PHCs are first registered (Figure 1). Thereafter, they visit the policlinic where in most cases a nurse or a TB worker (nurse with the responsibility to manage the TB program at PHC level) will take the anamnesis and perform a physical examination. Information obtained from anamnesis and the physical examination is recorded in the patient medical record. The National Tuberculosis Program (NTP) guidelines of Indonesia state that the presence of the general symptom cough for more than 3 weeks, or the presence of other symptoms such as blood in sputum, fever >1 month, loss of weight, dyspneu, chest pain, malaise, sweating at night and loss of appetite should raise suspicion of TB. Patients who are suspected of tuberculosis are referred to the laboratory for sputum examination. PRMs and PPMs have a laboratory that can do smear examination themselves. PSs will send the sputum sample to a PRM for examination. If the result of the sputum examination is positive, the patient will be counseled by the TB worker and receive treatment. Patients with a negative laboratory result will be sent back to the policlinic, receive treatment for their symptoms and be requested to come back after one week for a repeat sputum examination. Part of the patients will get a consultation with a medical doctor.

We performed a descriptive cross-sectional study from August 2004 to February 2005 in all 25 PHCs in Sidoarjo district to assess the quality of the diagnostic process. Data were collected about the knowledge of the health workers of TB, the knowledge about the information that should be provided to the patient on how to produce a good quality sputum sample, and the quality of smear examination. Also the availability of support services for the health center staff that help them in performing the activities for the TB program was assessed. The above information was collected through interviews with staff of PHCs. The quality of the laboratory diagnosis was assessed by providing 10 sputum slides obtained from the district laboratory with known result to the laboratory technicians for re-examination. The study population included the head of the PHC, the TB worker, a general nurse and the laboratory technician of each of the 25 PHCs.

A descriptive analysis was performed for quantitative data from interviews with health staff. Analysis was stratified by type of PHCs i.e. with and without microscope facilities. Statistical analysis using Chi-square and Fisher's exact tests was performed to compare knowledge of TB between nurses and TB workers. ANOVA was used to compare the characteristics of different types of health staff.

## Results

### Characteristics of the interviewed persons

A total of 100 health workers were interviewed. Sixty-four percent of the respondents were female (Table 1). The age of the respondents ranged between 24 and 59 with a mean age of 43.5 years. The heads of the PHCs (mean age 48.2 years, range 34–59) and the TB workers (mean age 45.9 years, range 34–55) were on average older than the general nurses (mean age 44.0 years, range 24–56) and the laboratory technicians (mean age 35.8, range 26–51) (ANOVA, F-value = 16.1 p < 0.001). The heads of the PHCs worked on average for 5.8 (range 1–16) years in the PHC, the laboratory technicians for 8.3 (range 1–30) years, the general nurses for 16.2 (range 1–32) years and the TB workers for 10.2 (range 2–33) years (ANOVA, F-value = 11.2 p < 0.001). Sixty percent of the nurses (general nurses and TB workers) had worked in PHC for 10 years or more.

**Table 1:** Characteristics of the respondents of the primary health centers in Sidoarjo district

**Characteristics**	**Type of respondent (%)**			
	
	**Head of PHC (n = 25)**	**TB worker (n = 25)**	**Nurse (n = 25)**	**Laboratory technician (n = 25)**
**Sex**				
Male	10 (40)	14 (56)	8 (32)	4 (16)
Female	15 (60)	11 (44)	17 (68)	21 (84)
**Age (in years)**				
≤30	0 (0)	0 (0)	1 (4)	0 (0)
31 – 40	1 (4)	7 (28)	7 (28)	6 (24)
41 – 50	15 (60)	10 (40)	10 (40)	15 (60)
>50	9 (36)	8 (32)	7 (28)	4 (16)
**Duration of work (in years)**				
>20	0 (0)	2 (8)	7 (28)	1 (4)
10 – 20	3 (12)	8 (32)	13 (52)	7 (28)
5 – 9	15 (60)	12 (48)	4 (16)	8 (32)
<5	7 (28)	3 (12)	1 (4)	9 (36)

PHC, Primary Health Centre; TB, Tuberculosis

All heads of PHCs had been trained as a medical doctor. Three (12%) general nurses were trained as nurse assistant, 16 (64%) received training at the nursing school and 6 (24%) at the nursing academy. TB workers were trained at the nursing school (72%) and at the nursing academy (28%). Eighteen (72%) laboratory technicians had received specific education for laboratory work, 14 (56%) at the school for laboratory technicians and 4 (16%) at the academy for laboratory technicians. Seven (28%) had not received special education for laboratory work.

### Knowledge of TB

Knowledge of TB workers and general nurses about TB symptoms was comparable (Table 2). Only night sweats were more frequently mentioned by the TB workers (p = 0.02). Although all TB workers had received TB training, 16% did not mention 'cough more than three weeks' as general symptom of TB. Most (96%) general nurses did not receive training on TB, even though 80% of them mentioned 'cough more than three weeks' as a general symptom of TB.

**Table 2:** Comparison of knowledge of nurses and TB workers at the primary health center in Sidoarjo district

**Knowledge of TB**	**Nurse (%) (n = 25)**	**TB worker (%) (n = 25)**	p-value*
**Symptoms**			
Cough >3 weeks	20 (80)	21 (84)	n.s
Sputum with blood	14 (56)	18 (72)	n.s
Fever	4 (16)	9 (36)	n.s
Weight loss	12 (48)	19 (76)	n.s
Dyspneu	16 (64)	19 (76)	n.s
Chest pain	4 (16)	7 (28)	n.s
Malaise	6 (24)	4 (16)	n.s
Night sweats	8 (32)	17 (68)	0.023
Loss of appetite	17 (68)	16 (64)	n.s
**Cause of TB: *Mycobacterium tuberculosis***	10 (40)	23 (92)	<0.001
**Complications of TB**			
Hemophthysis	2 (8)	5 (20)	n.s
Collapse of the lung	0 (0)	0 (0)	-
Bronchiectasis	1 ((4)	1 (4)	n.s
Pneumothorax	0 (0)	2 (8)	n.s
Disseminated TB	2 (8)	1 (4)	n.s
Cor pulmonary	0 (0)	0 (0)	-
**Duration of infectiousness after start of TB treatment**	0 (0)	19 (76)	<0.001

* n.s. = not significant (Fishers exact test p-value > 0.05)

TB, Tuberculosis;

The cause of TB (i.e. *Mycobacterium tuberculosis *infection) was known by 40% of the general nurses and 92% of the TB workers (p < 0.001). The two TB workers who did not mention *M. tuberculosis *as the cause of TB mentioned that bacillus or TB bacteria caused TB.

Eighty percent of the nurses and TB workers considered an individual to be suspected of TB when the general symptom cough for more than 3 weeks, or one of the other symptoms were present. The other nurses and TB workers based suspicion only on other symptoms such as sputum with blood.

Less than half of the general nurses (44%) and TB workers (40%) knew the requirements for diagnosing pulmonary TB according to the national guidelines i.e. two or more sputum samples positive for AFB or one sputum sample positive for AFB and a chest X-ray with pathology consistent with TB or negative sputum samples and a chest X-ray with pathology consistent with TB.

Hemoptysis (coughing up blood) was the complication of TB most often mentioned by the interviewed general nurses (8%) and TB workers (20%). Other known complications mentioned in the NTP guidelines such as collapse of the lung, bronchiectasis, pneumothorax, disseminated TB, and cor pulmonale were mentioned by only one or two nurses or TB workers.

None of the general nurses knew that the duration of infectiousness of TB after the start of TB treatment is approximately two weeks. They mentioned durations of infectiousness from one month to three months or they did not know the duration. Most TB workers (76%) knew the duration of infectiousness of TB after the start of treatment. Five TB workers mentioned duration of infectiousness of one month to three months.

### Health education for providing sputum sample

During the first contact with a TB suspect the health worker is supposed to give health education about tuberculosis, the technique of producing a good sputum sample, to provide suggestions in case the TB suspect has problems with producing sputum and to provide information at what time the TB suspect should produce the samples (spot, morning, spot).

Most general nurses (76%) and TB workers (84%) provided health education about at least some aspects of sputum production (Table 3). Twelve (48%) general nurses and 13 (52%) TB workers provided information about how to produce sputum. Nine (36%) general nurses and 17 (68%) TB workers provided information about the use of expectorant (p = 0.046). Eight (32%) general nurses and 11 (44%) TB workers reported to inform the TB suspect about the time of the day to produce sputum. Thirty-six percent of the TB workers provided information about all three topics, whereas only 20% of the general nurses reported to provide all information.

**Table 3:** Health education for sputum collection to TB suspects by general nurses and TB workers in health centers with microscope facilities and without microscope facilities

**Health education given by the nurses and TB workers to TB suspects**	**Type of primary health center**			
	
	**With microscope facility**		**Without microscope facility**	
	
	**Nurse n = 11**	**TB worker n = 11**	**Nurse n = 14**	**TB worker n = 14**
**Provided information about how to produce sputum**	9 (82)	9 (82)	10 (71)	12 (86)
**Type of information**				
Technique to produce sputum	3 (27)	1 (9)	4 (29)	2 (14)
Information about expectorant	2 (18)	2 (18)	2 (14)	5 (36)
Time to produce sputum	1 (9)	1 (9)	2 (14)	0 (0)
Combination of above	3 (27)	5 (45)	2 (14)	5 (36)

TB, Tuberculosis

Thirteen (52%) laboratory technicians provided health education for sputum examination (Table 4). They mentioned that an explanation is important because sputum examination is essential for the correct diagnosis of TB patients (32%), because it motivates TB suspects to provide sputum (16%), or for other reasons (4%). Information given by laboratory technicians consisted of the reason for sputum examination, use of expectorant if there were problems with the production of sputum, and what to do if the suspect still had difficulty to produce sputum. Twenty-four (96%) laboratory technicians suggested unorthodox methods for stimulating sputum production such as drinking hot tea, running, jumping and sun bathing.

**Table 4:** Health education for sputum production to TB suspects by laboratory technicians in primary health center with microscope facilities and without microscope facilities

**Type of health education laboratory technicians would give to TB suspects**	**Type of primary health center (%)**		
	
	**With microscope, PRM and PPM (n = 11)**	**Without microscope, PS (n = 14)**	**Total (n = 25)**
**Explain about sputum examination**	4 (36)	9 (64)	13 (52)
**Frequency of explaining about sputum examination**			
Always	2 (18)	8 (57)	10 (40)
Sometimes	2 (18)	1 (7)	3 (12)
**Information provided to patient who has problems producing sputum**			
Hot tea & expectorant	6 (55)	2 (14)	8 (32)
Hot tea	4 (36)	9 (64)	13 (52)
Expectorant	0 (0)	1 (7)	1 (4)
Other (Running, jumping, sunbathing)	1 (9)	2 (14)	3 (12)
**What do you suggest the patient if (s)he cannot produce sputum**			
Come to policlinic	5 (45)	8 (57)	13 (52)
Try again	5 (45)	6 (43)	11 (44)
Don't know	1 (9)	0 (0)	1 (4)

TB, Tuberculosis; PRM, Puskesmas Rujukan Mikroskopis (laboratory referral health center); PPM, Puskesmas Pelaksana Mandiri (independent laboratory health center); PS, Puskesmas Satelit (satellite health center)

### Quality of smear examination

The quality of the smear examination was assessed by having the laboratory technicians of the 11 health centers that performed sputum examination read 10 slides with a known result (slides were provided by the district laboratory). Fifty-five percent correctly identified all positive slides as positive (Table 5). Five (45%) of the 11 laboratory technicians correctly identified 100% of the negative slides as negative. Two PHCs (18%) identified less than 75% of the positive slides correctly and three PHCs (27%) identified less than 75% of the negative slides correctly. Of the 60 positive slides provided to the 11 laboratories 11 (18%) were not identified as such and of 50 negative slides 8 (16%) were identified as positive.

**Table 5:** Quality of smear examination in 11 health care facilities with a laboratory in Sidoarjo district

**Number**	**Type**	**Number of positive slides provided**	**Number of negative slides provided**	**Number of positive slides identified**	**Number of negative slides identified**	**Sensitivity**	**Specificity**
1	PRM	8	2	4	2	50	100.0
2	PRM	4	6	4	4	100	66.7
3	PRM	4	6	3	6	75	100.0
4	PRM	8	2	6	2	75	100.0
5	PRM	8	2	5	2	62.5	100.0
6	PRM	4	6	4	5	100	83.3
7	PRM	4	6	4	4	100	66.7
8	PRM	4	6	4	5	100	83.3
9	PRM	8	2	7	1	87.5	50.0
10	PPM	4	6	4	6	100	100.0
11	PPM	4	6	4	5	100	83.3

Total						86.4	84.8

PRM, Puskesmas Rujukan Mikroskopis (laboratory referral health center); PPM, Puskesmas Pelaksana Mandiri (independent laboratory health center)

In most health care services it took two to seven days to complete the sputum examination. In PSs this was longer for 33% of the samples.

### Support in PHC for the TB diagnostic process

TB workers and laboratory technicians need TB forms (Form 01, 02, 04, 05 and 06) and laboratory materials to be able to perform their work. All TB workers reported that the forms were available and only one general nurse reported that there had been shortages of TB forms. Shortage of laboratory materials was reported by one laboratory technician. In 12 (48%) PHCs laboratory materials were ordered 2 to 4 times per year. In the other PHCs materials were ordered when needed.

According to the majority of the heads of PHCs (72%) there was no specific budget for TB control in the PHC. The general budget provided by the district and province was used to buy TB forms and laboratory equipment. There was no specific budget available for activities such as community health education, active case finding, transportation fee for staff and patients, and extra food for patients. Only 2 (8%) heads of PHCs considered the budget available for the TB program sufficient. Eight (32%) PHCs had budget available for incentives for staff working in the TB program.

Most heads of PHCs (96%) were of the opinion that there was a sufficient number of staff for suspect screening. The number of staff for laboratory diagnosis was considered insufficient by 8 (32%) of them. Only one general nurse had received training in TB control. All TB workers had received training in TB control, 10 (40%) of them once, 11 (44%) twice, and 4 (16%) TB workers received training three times. Seventeen (68%) laboratory technicians had received specific training. Of the 14 laboratory technicians working in the laboratory of a PS 8 (57%) had never been trained although they were responsible for collection of sputum samples, and preparation and fixation of slides. Fifty-two percent of the heads of PHCs considered the performance of the staff working in the TB program not optimal. The main reason mentioned was the fact that not all staff was properly trained. Trainings that were needed according to them were in the field of communication skills, organization and management, and quality assurance. Sixty percent of the heads of PHC considered especially TB training for general nurses and midwifes important because most trainings up to now focused on TB workers and laboratory technicians.

The job of the wasor is to supervise the activities for TB control in the PHCs. All TB workers were visited 3–4 times per year by the wasor. In most cases (96%) the wasor gave advice to the TB worker. Inspection of form 01, 04, 05 and 06 was performed in 12% of the supervision visits. Also the laboratory technicians reported that they were frequently (4× per year) supervised by the wasor.

All TB workers at the PHC were responsible for more than one program (policlinic, public health nursing, integrated health post services [posyandu] etc.). Most of them (80%) were responsible for three or more programs inside and outside the PHC. All but two had duties outside the PHC. TB workers reported that their workload was quite high.

The general nurses in the PHC have to examine and diagnose all patients who present themselves to the PHC. Besides this work they were also responsible for programs such as posyandu, public health nursing, communicable disease programs e.g. malaria, Dengue Haemorrhagic Fever (DHF), and leprosy. Eleven (44%) nurses were responsible for one or two programs and 14 (56%) for three or more programs. Also the nurses reported a high workload. Training of staff or re-allocation of staff was difficult according to the heads of PHC, because staff was already overloaded by other programs and because of staff shortages.

In total, 56% of the laboratory technicians were involved in other programs besides the TB program. Only 2 (18%) of those working in the PRMs and PPMs were involved in other programs and almost all (86%) in the PSs. In most cases they were involved in programs outside the primary health center such as the school health program, posyandu, and the national immunization program.

Fifteen (60%) TB workers reported collaboration with the general nurses and the laboratory technicians for the TB program whereas all general nurses and laboratory technicians reported to collaborate with the TB worker. The majority (84%) of the TB workers said that there were no problems in collaborating with the other staff in the PHC. Meetings about the TB program were organized in 72% of the PHC. The frequency of the meetings was low, often only once a year. The head of the PHC had an important role in the meetings, especially in providing solutions for the identified problems.

## Discussion

The requirements for optimal identification of TB suspects and diagnosis of TB patients in a PHC setting (knowledge of health care workers of TB symptoms, providing health education for TB suspects about how and why to submit good quality sputum samples, and high quality sputum smear examination) were not present in all PHCs in Sidoarjo district. This may be the reason for the low case detection rate in the district. The main identified problem was the low number of general nurses and laboratory technicians that had received training in TB control resulting in lack of knowledge about identification of TB suspects, inadequate health education for sputum collection, and insufficient quality of the laboratory diagnosis. Furthermore, the reported high workload may influence the quality of the diagnostic process.

Most general nurses and TB workers knew that cough for more than 3 weeks can be a symptom of tuberculosis. Other symptoms often related to TB were not so well known. In general, the TB workers who had received training in TB control mentioned more symptoms of TB than the general nurses who had not received training in TB control. Also knowledge about the cause of TB and the duration of infectiousness after start of treatment was better in TB workers. Knowledge about the most important symptom for identification of TB suspects (cough >3 weeks) was less good in our study compared to knowledge of health workers in Vietnam (98% knew most common symptom of TB) and health staff in PHC in Iraq (93% mentioned cough >3 weeks) [7,8]. Also knowledge about the cause of TB was not as good in our study (40% and 92%) compared to the studies in Vietnam and Iraq. In a study in India among nurses of the TB institute and of a large hospital, knowledge of the cause of TB was comparable to that in our study with a considerable number of nurses mentioning a virus as the causative organism [9].

Obtaining a good quality sputum sample is essential for the diagnosis of TB. It has been shown that addressing the importance of sputum examination and instructing suspects how to produce adequate samples increases the quality of the sputum sample and the detection rate [10]. Health education information provided for sputum collection was not complete in our study sample and contained information outside of the guidelines. Thus better training of health workers in instructing patients may provide better quality sputum samples and a higher yield of sputum smear positives.

With 18% false negatives and 16% false positives the performance of the laboratory technicians of the eleven health centers that performed sputum examination can be improved. The rates are high compared to other studies, i.e. the quality assessment results of rural health units in Philippines showed a false positive rate of 5.5% and in a district hospital laboratory in Malawi both false negative and false positive rates were less than 2% [11,12]. In these areas laboratory quality control had been introduced. The high false negative rate gives that smear positive TB patients do not get appropriate treatment, whereas the high false positive rate gives that patients receive treatment unnecessarily. Since only a limited number of slides were used to assess the quality of the laboratory a more comprehensive evaluation of the laboratory is needed and introduction of rigorous supervision and a quality control system.

The general education of nurses seemed to be sufficient. However, only one general nurse had received specific training on TB in contrast to all TB workers. This may hamper the identification of TB suspects by general nurses. Furthermore, since they did not receive training in how to instruct suspects to provide a good quality sputum sample also sputum collection may be of less quality. Since the general nurse is first in line to examine patients and thus to identify TB suspects training of these nurses in recognizing symptoms of TB and providing health education for sputum collection is necessary. A quarter of the laboratory technicians had not received adequate general training in laboratory work and only 17 of the 25 had received specific training for TB diagnosis. This lack of training may explain the poor results of the quality control evaluation of the laboratory.

According to the Sidoarjo health office there is an adequate budget for TB control (Sidoarjo Health Office, 2003). The majority of the heads of the PHCs reported that there was no specific budget for TB control in the PHC and only 2 heads of PHCs considered the budget available for the TB program sufficient. Also, there was no specific budget available for activities such as community health education, active case finding, transportation fee for staff and patients, and extra food for patients. The results of the interviews with the heads of the PHCs thus show a contradiction with the opinion of the district health authorities. A discussion between the heads of the PHCs and the district health authorities on this may help in getting an adequate budget for TB control.

This study was performed in Sidoarjo district. Therefore, the results are only applicable to Sidoarjo district. Since the organization of primary health care is comparable in other districts and provinces in Indonesia it is likely that an assessment of the quality of the diagnostic process in other districts will yield comparable findings.

The quality of the diagnostic process was assessed by interviews with key staff in the health care facility. It is possible that the health workers provided socially desirable answers instead of the correct answers. A study in which the actions of the health workers are observed may prevent this. However, the presence of an observer is also likely to change the behavior of the health worker.

## Conclusion

The quality of the diagnostic process for tuberculosis at PHC in Sidoarjo district should be improved on all levels, i.e. identification of TB suspects, collection of sputum samples, and examination of sputum samples. Training in TB control of all general nurses and the laboratory technicians that have not received training would be a good first step to enhance diagnosis of TB and to improve the case detection rate. The quality of the laboratory diagnosis should be more thoroughly assessed and if necessary improved.

## Abbreviations

CDC, Communicable Disease Center; CDR, Case Detection Rate; DHF, Dengue Haemorrhagic Fever; NTP, National Tuberculosis Program; PHC, Primary health care centre; PPM, Puskesmas Pelaksana Mandiri (independent laboratory health center); PRM, Puskesmas Rujukan Mikroskopis (laboratory referral health center); PS, Puskesmas Satelit (satellite health center); TB, Tuberculosis

## Competing interests

Sri Yuliwati is employed by the district health office of Sidoarjo district that covers the study area. Since we do not expect that the district health office will gain or lose financially from this publication we find him not to have competing interests. None of the other authors of the manuscript had any financial or non financial competing interests.

## Authors' contributions

The study was performed and the manuscript was developed as part of an operational research course. B, LDR, MS, TR, D, and SY participated in the course. CUW was the facilitator of the course and MJW was the international facilitator. B, LDR, MS, TR, D, and SY developed the study protocol, performed the data collection, data entry and data checking, and did the analysis and report writing assisted by CUW and MJW. The manuscript was prepared by B, LDR, MS, TR, D, SY and CUW and revised by MJW. All authors read and approved the final manuscript.

## Pre-publication history

The pre-publication history for this paper can be accessed here:


